# Potentials of participatory approaches for urban mental health

**DOI:** 10.1093/eurpub/ckac129.396

**Published:** 2022-10-25

**Authors:** A Wasmer, H Müller, J Rehn-Groenendijk

**Affiliations:** 1 Civil Engineering, Darmstadt University of Applied Sciences, Darmstadt, Germany; 2 Social and Cultural Sciences and Social Work, Darmstadt University of Applied Sciences, Darmstadt, Germany; 3 Design Research, Darmstadt University of Applied Sciences, Darmstadt, Germany

## Abstract

Aspects of mental health, society, space and environment share entangled relations being studied in health geography. Recreational spaces as well as places that are commonly perceived as strenuous, unsafe, or highly stressful are unevenly distributed within urban areas, which is also associated with spatial differences in mental disorders. Spaces in general represent social constructions that reflect power inequalities; they are filled with subjective emotional resonances and sometimes visualize stigmatization of specific groups. As such, the interplay of socio-demographic factors, socio-economic factors and built environment is complex. To capture these entanglements represented in heterogeneous user groups, participatory approaches promise valuable insights. Yet, despite their great potential for fostering mental health in urban space, participatory approaches are still less common in health geography. Therefore, critical voices question whether the limitations of marginalized groups have been sufficiently considered in this field of study so far. Similar challenges arise in urban planning processes: Specific (vulnerable) groups such as children, women, foreign residents, and people with disabilities or elderly people are insufficiently included in planning processes, leading to an underrepresentation of their needs in the resulting environments. To tackle this shortcoming, the approach of co-creation offers a process in which participants jointly develop a solution without being the object of research or interview partners, but creators. Using rather practical or creative (e.g., joint mapping of the built environment, photo-elicitation) than discursive techniques allows contributions from population groups otherwise often excluded from planning processes. Despite certain limitations, participatory approaches promise the possibility to develop appropriate and just solutions in urban mental health.

